# Intraoperative ultrasonographic evaluation of inferior orbital rim reduction in non-comminuted zygomatic fractures: A case series of six patients

**DOI:** 10.1016/j.ijscr.2025.111771

**Published:** 2025-08-05

**Authors:** S. Morand, E. Lange, A. Lari, B. Alali, F. Almukaimi, A. Gleizal

**Affiliations:** a MaxilloFacial Surgery Department, Hospices civils of Lyon, Croix Rousse Hospital, 103 Grande rue de la Croix Rousse, 69004 Lyon, France; b Plastic and Reconstructive Surgery Department, Al-Babtain Center for Burns and Plastic Surgery, Sabah health Region, Kuwait

**Keywords:** Zygomatic fracture, Intra-operative ultrasonography, Orbital rim, Case report

## Abstract

**Introduction and importance:**

Zygomatic fractures are common in maxillofacial trauma and often require surgical intervention for the restoration of facial symmetry. The inferior orbital rim significantly influences cheekbone asymmetry. The management of zygomatic fractures involves estimating the adequacy of reduction by comparing the malar projection on the fracture and healthy sides. This clinical approach is subjective and lacks precision.

**Presentation of case:**

Six consecutive patients with non-comminuted zygomatic fractures were included in the study. Ultrasound was performed pre- and post-reduction during the surgery to visualize the fracture site and measure the displacement by visualizing the periosteal discontinuity. The results were compared with those of pre- and post-operative CBCT scans.

**Discussion:**

Ultrasound demonstrated comparable efficacy to CBCT for assessing fracture reduction. Unlike CBCT, ultrasound involves no radiation exposure and allows for real-time intra-operative assessment, potentially reducing the need for revision surgery.

**Conclusion:**

Ultrasound is a precise and practical modality for evaluating zygomatic fracture reduction during J-hook procedures*.* It offers a radiation-free, inexpensive and readily available modality that improves intra-operative decision-making.

## Highlights

• Intraoperative ultrasonography enables precise, radiation-free assessment of zygomatic fracture reduction, specifically at the inferior orbital rim.
• Compared to CBCT, ultrasound delivers equivalent accuracy in detecting bone displacement and verifying periosteal continuity during surgery.
• Real-time imaging supports intraoperative decision-making, potentially reducing the need for secondary revision procedures.
• This is among the first case series to document ultrasound-guided evaluation of orbital rim reduction during J-hook intervention.
• The study highlights ultrasonography as a practical and reproducible tool in maxillofacial trauma surgery.

## 1 Introduction

Zygomatic fractures represent a significant portion of maxillofacial fractures, accounting for approximately 39 % [1]. There are three main types of zygoma fractures according to the Zing classification [2,3]. Types A and B (non-comminuted fractures) are amenable to closed reduction with a J-hook, while complex fractures necessitate open surgery. The management of zygomatic fractures involves estimating the adequacy of reduction by comparing the malar projection on the fracture and healthy sides. Additionally, palpation of the inferior orbital rim allows the detection of persistent bone gaps. This clinical approach is subjective and lacks precision. Patients often complain of insufficient cheekbone projection and facial asymmetry. Postoperative control imaging generally involves cone-beam computed tomography (CBCT) or radiographs, which offer good precision but require radiation exposure.

The interest of using medical imaging to control in real time the fracture reduction and avoid the risk of secondary surgery was demonstrated by some authors [4].

While some authors have described the use of intraoperative scanning, it is time consuming and requires advanced equipment [5].

Ultrasonography allows reduction control by identifying periosteum overlying the cortices, allowing visualization of bony structures without radiation exposure. It offers several advantages, including a high precision, with measurements to the tenth of a millimeter, widespread availability, ease of use, and reduced time. This study presents a series of six type-B zygomatic fractures that underwent ultrasound control of inferior orbital rim reduction following percutaneous J-hook reduction.

This case series has been reported in line with the SCARE checklist [6], and used the PROCESS guideline revised in 2025 [7].

## 2 Case series

This study aimed to control the reduction of lower orbital rim following J-hook reduction of the zygomatic tripod in six consecutive patients. The precision of ultrasound control was compared with that for CBCT.

The inclusion criteria were patients aged ≥18 years who consulted between September 2023 and February 2024 with zygomatic tripod fractures and were eligible for J-hook reduction (i.e., non-comminuted fractures). The exclusion criteria were comminuted zygomatic fractures and those with a large intermediate fragment in the lower orbital rim. Five men and one woman were included in the study. All patients gave written consent to the study.

Preoperative CBCT was used to diagnose the zygomatic tripod fractures, with fracture displacement defined as a depression in the zygomatic body on axial sections. The height of sagittal displacement (millimeters) was measured directly on the axial sections of CBCT using the ruler tool.

**Fig. 1:**
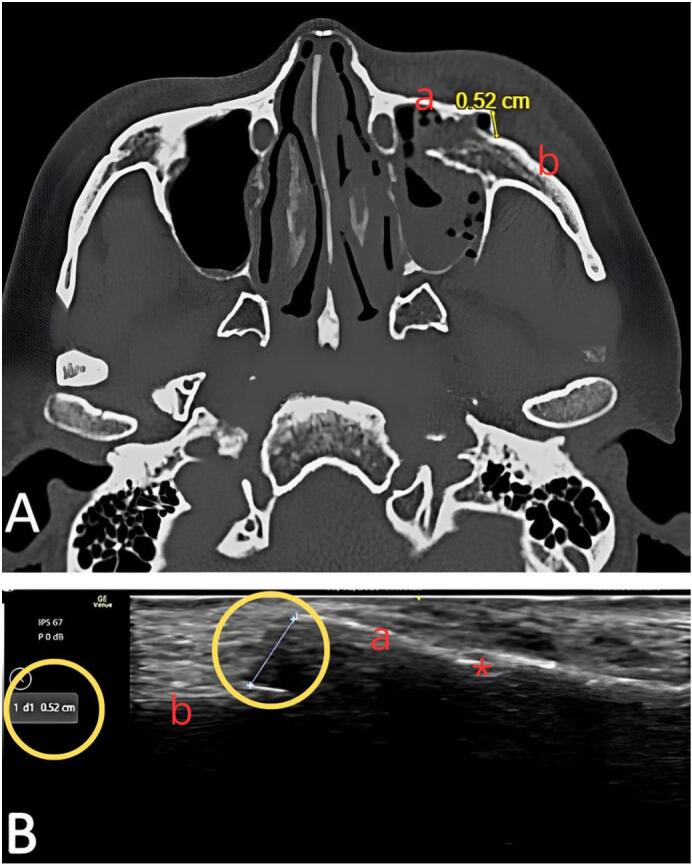
Intraoperative photography. Real time assessment of a zygomatic fracture reduction by ultrasonography. a: J-hook inserted below the body of the zygoma. b: ultrasound probe. c: image visualization.

The patients were scheduled for J-hook reduction under general anesthesia and nasotracheal intubation. Only one Maxillo-Facial surgeon with more than 3 years of experience in the specialty performed ultrasonography. An ultrasound was performed before the reduction (GE Healthcare Venue Go™, L4-12t probe, depth: 5.0 cm). The probe was placed on the zygomatic arch, which is easily palpable and constituted the starting point, with its axis parallel to the axis of the zygomatic arch. It was then moved along the zygomatic arch in a forward direction, following the inferior orbital rim until the fracture line was visualized **(**Fig. 1**).** A screenshot was taken at this location and the distance between each bone fragment was measured in millimeters, representing the extent of displacement.

After obtaining the measurements, the fracture was reduced using a J-hook introduced percutaneously under the body of zygoma. The skin was marked at the junction of a vertical line passing through the external canthus and a horizontal line passing through the ala of the nose. The J-hook was positioned and traction was applied in forward and outward directions to reduce the fracture. The surgeon's non-dominant hand was positioned at the lower orbital rim fracture site to control the reduction.

Following the reduction, another ultrasound was performed following the previously described method to confirm lower orbital rim continuity and the absence of a gap between the fracture ends.

If the ultrasound showed good fracture reduction, cheekbone symmetry was assessed clinically and a forced duction test (assess passive movement of the globe upward) was used to confirm the absence of lower rectus muscle entrapment, thus completing the intervention.

Postoperatively, the patients were monitored for one night in the hospital. On postoperative day 1, the patient underwent CBCT to confirm the results of intraoperative ultrasonography. All patients underwent CBCT before the surgery, intraoperative ultrasonography before and after the reduction, and CBCT on the first post-operative day. None of the six patients presented any postoperative complications.

The extent of the fracture displacement was measured using both CBCT and ultrasound, and the values were compared. After the surgical intervention, the reduction was evaluated using both ultrasound and postoperative CBCT, allowing a comparison of the two modalities.

**Fig. 2:**
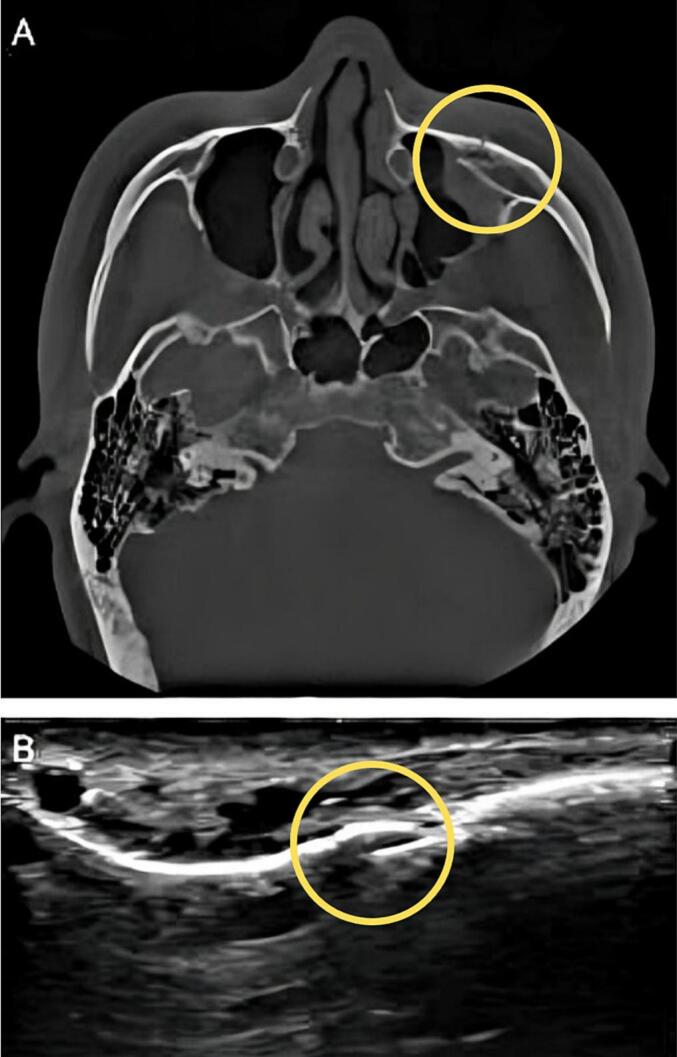
A: CBCT measurement of orbital rim displacement. a: medial fragment of orbital rim. b: lateral fragment of orbital rim. B: Ultrasonographic measurement of orbital rim displacement for the same patient. a: medial fragment of orbital rim. b: lateral fragment of orbital rim. star: infraorbital foramen. Yellow circle: fracture site and measurement of fracture displacement. (For interpretation of the references to colour in this figure legend, the reader is referred to the web version of this article.)

**Fig. 3:**
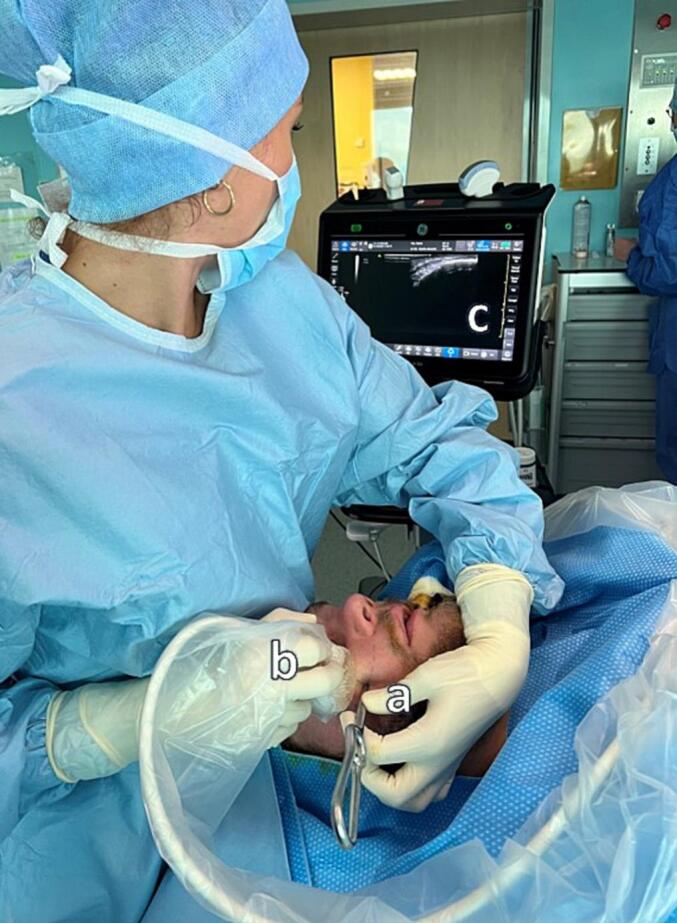
A: CBCT visualization of orbital rim reduction. B: Ultrasonographic visualization of orbital rim reduction. Yellow circle: fracture site. (For interpretation of the references to colour in this figure legend, the reader is referred to the web version of this article.)

For the preoperative evaluation of fracture displacement, both CBCT and ultrasound had similar precision levels **(**Fig. 2**)**. After fracture treatment, ultrasound confirmed good fracture reduction by allowing visualization of periosteal continuity in the malar bone, with similar results obtained using postoperative CBCT **(**Fig. 3**)**.

## 3 Results

For the six cases studied, ultrasound allowed fracture site visualization and measurement of the displacement with the same precision as CBCT **(**Table 1**)**. It also proved useful for verifying the reduction by highlighting the periosteal continuity during the same operating time.

**Table 1:** Inferior orbital rim displacements measured in millimeters, using ultrasound and cone-beam computed tomography.

Patient	Ultrasound preoperative (mm)	Ultrasound postoperative (mm)	CBCT preoperative (mm)	CBCT postoperative (mm)
1	44	0	43	0
2	26	0	27	0
3	62	0	62	0
4	35	0	34	0
5	52	0	52	0
6	51	0	50	0

## 4 Discussion

These six clinical cases demonstrate the efficacy of ultrasound to assess inferior orbital rim fracture reduction in the context of zygomatic fractures treated through J-hook reduction.

The use of ultrasonography to diagnose a zygomatic fracture was already been reported [8] and demonstrated a better reliability than radiography.

Singh et al. previously reported the use of ultrasound in closed reduction of zygomatic arch fractures [9]. Buller et al. compared the reduction of zygomatic arch fractures using ultrasound with the conventional blind method, and reported a significant reduction in the mean persistent angle of displacement compared to the control group [10]. However, to the best of our knowledge, the reduction of inferior orbital rim has not been investigated previously even though its displacement contributes significantly to cheekbone asymmetry.

In the present study, ultrasound allowed fracture site visualization and measurement of the displacement with the same precision as CBCT **(**Table 1**)**. It also proved useful for verifying the reduction by highlighting the periosteal continuity.

For assessing zygomatic fracture reduction using J-hook, ultrasound showed an efficacy similar to CBCT but without any radiation exposure [11]. The mean radiation exposure for a CBCT varies from 0.2 mSv to 0.7 mSv, which is non-negligible [12]. Moreover, if the fracture necessitates to be revised because of a persistent displacement, a second control by CBCT is needed and the radiation exposure increase. The time required for the installation of equipment, measurement of displacement, and the control of reduction increased the operating time by approximately 15 min. This tool is easy and safe to use, without any risk of operating field contamination, as the location of the probe is distant from the insertion site of the J-hook, and the probe is protected by a sterile cover.

Clinical examination alone is unreliable for intraoperative assessment of reduction because of several factors, such as the presence of edema. Despite apparent cheekbone symmetry on intraoperative examination, if the ultrasound shows persistent discrepancy between the bone fragments, the surgeon can act during the same general anesthesia to surgically correct the problem. This avoids the need for surgical revision due to insufficient reduction on subsequent examinations and CBCT.

A disadvantage of ultrasound is that is done in two dimensions, and there is no possibility of three-dimensional reconstruction compared to CT scanning. Particularly, it gives a degree of displacement in the sagittal plane, not in the transversal plane and frontal plane. Consequently, ultrasonography doesn't allow to control the fracture reduction in both transversal and frontal plane. Nevertheless, the most important direction to correct is the antero-posterior direction, as it determines the malar projection, and the aesthetic result.

Manipulating the ultrasound probe to obtain the most clear and legible image necessitates a short learning by the surgeon, particularly concerning the degree of pression to apply on the cutaneous and sub-cutaneous tissue allowing ultrasound penetration. This learning could be done fast and easy.

The sample size of this study limited its strength, but the results encourage a future study with a larger number of patients.

## 5 Conclusion

The study showed that ultrasound is a precise, reproducible and practical modality for evaluating zygomatic fracture reduction during J-hook procedures. It offers a radiation-free, inexpensive and readily available modality that may reduce fracture reduction errors rates and improve intra-operative decision-making. Further prospective studies with a larger number of patients are required to compare inferior orbital rim reduction with and without ultrasound.

## Author contribution

Sanela Morand: Writing, Operator

Edouard Lange, Aqeel Alari, Ahmad Alali, Fahad Almukaimi: Data collection

Arnaud Gleizal: Contribution, Validation

## Consent

Written informed consent was obtained from all patients for publication of this case series and accompanying images. A copy of the written consent is available for review by the Editor-in-Chief of this journal on request.

## Ethical approval

In accordance with French law, it is a retrospective study, data collected were anonymous. The confidentiality policy was fulfilling with European General Data Protection Regulation. Review by an ethics committee was not required.

## Guarantor

Sanela Morand

## Sources of funding

The authors declare that they have no sources of funding.

## Declaration of competing interest

The authors declare that they have no known competing financial interests or personal relationships that could have appeared to influence the work reported in this paper.
